# Targeting the HGF-cMET Axis in Hepatocellular Carcinoma

**DOI:** 10.1155/2013/341636

**Published:** 2013-03-31

**Authors:** Neeta K. Venepalli, Laura Goff

**Affiliations:** ^1^Division of Hematology-Oncology, University of Illinois at Chicago, 840 South Wood Street, Suite 820-E, MC 713, Chicago, IL 60612, USA; ^2^Hematology/Oncology Fellowship Program, Division of Hematology-Oncology, Vanderbilt Ingram Cancer Center, 777 Preston Research Building, Nashville, TN 37232-6307, USA

## Abstract

Under normal physiological conditions, the hepatocyte growth factor (HGF) and its receptor, the MET transmembrane tyrosine kinase (cMET), are involved in embryogenesis, morphogenesis, and wound healing. The HGF-cMET axis promotes cell survival, proliferation, migration, and invasion via modulation of epithelial-mesenchymal interactions. Hepatocellular cancer (HCC) is the third most common cause of worldwide cancer-related mortality; advanced disease is associated with a paucity of therapeutic options and a five-year survival rate of only 10%. Dysregulation of the HGF-cMET pathway is implicated in HCC carcinogenesis and progression through activation of multiple signaling pathways; therefore, cMET inhibition is a promising therapeutic strategy for HCC treatment. The authors review HGF-cMET structure and function in normal tissue and in HCC, cMET inhibition in HCC, and future strategies for biomarker identification.

## 1. Introduction

Hepatocellular carcinoma (HCC) is the sixth most common malignancy worldwide and the third most common cause of global cancer related mortality [1, 2]. HCC burden disproportionately impacts developing countries and males; as of 2008, 85% of cases occurred in Africa and Asia, with worldwide male: female sex ratio of 2.4 [2]. Risk factors for the development of HCC include chronic liver inflammation from hepatitis B and C infection, autoimmune hepatitis, excessive alcohol use, nonalcoholic steatohepatitis, primary biliary cirrhosis, environmental carcinogens such as aflatoxin B, and genetic metabolic disease (such as hemochromatosis and alpha-1 antitrypsin deficiency). Prognostic and therapeutic options are dependent upon the severity of underlying liver disease, and median overall survival (OS) for metastatic or locally advanced disease is estimated at 5–8 months. HCC is relatively refractory to cytotoxic chemotherapy, likely due to overexpression of multidrug-resistant genes [3], protein products such as heat shock 70 [4] and P-glycoprotein [5], and p53 mutations. Presently, systemic therapeutic options in the locally advanced or metastatic setting are limited to sorafenib, an oral multikinase inhibitor targeting Raf kinase, vascular endothelial growth factor (VEGF), and platelet-derived growth factor (PDGF) receptor tyrosine kinase signaling. 

Although the transition from normal hepatocyte to HCC is not fully understood, hepatocarcinogenesis is a complex multistep process driven by accumulation of heterogeneous molecular alterations from initial hepatocyte injury to metastatic invasion. Inflammation results in hepatocyte regeneration, which induces fibrosis and cirrhosis through cytokine release. Dysplastic nodules subsequently progress to early HCC through cumulative genetic alterations, while advanced HCC often involves intrahepatic metastasis and portal vein invasion. Molecular alterations implicated in HCC development include mutations in oncogenes and tumor suppressor genes (p53 and p16), epigenetic alterations, chromosomal changes, and aberrant activation of signaling cascades necessary for proliferation, angiogenesis, invasion and metastasis, and survival. Pathogenesis of early and advanced HCC may be modulated through different mechanisms; for example, p53 mutations, p16 gene silencing, and aberrant AKT signaling are more frequently observed in advanced HCC [4–6]. The molecular pathogenesis of HCC is multifactorial and is reliant upon dysregulation of multiple pathways including WNT/b-catenin, mitogen-activated protein kinase (MAPK), phosphatidylinositol-3 (PI3K)/AKT/mammalian target of rapamycin (mTOR), VEGF, PDGF, insulin-like growth factor (IGF), epidermal growth factor (EGF), TGF-beta, and hepatocyte growth factor [6, 7]. 

The hepatocyte growth factor (HGF) and its transmembrane tyrosine kinase receptor, cellular MET (cMET) promote cell survival, proliferation, migration, and invasion via modulation of epithelial-mesenchymal interactions. HGF-cMET signaling is critical for normal processes such as embryogenesis, organogenesis, and postnatal tissue repair after acute injury. HGF-cMET axis activation is also implicated in cellular invasion and metastases through induction of increased proliferation (mitogenesis), migration and mobility (motogenesis), three-dimensional epithelial cell organization (morphogenesis), and angiogenesis.

## 2. HGF-cMET Axis

HGF was first discovered in 1984 as a mitogenic protein for rat hepatocytes *in vitro* [8]. HGF was subsequently found to be indistinguishable from scatter factor, a fibroblast-derived motility factor promoting epithelial cell dispersal [9] and three-dimensional branching tubulogenesis [10]. HGF is secreted primarily by mesenchymal cells (or by stellate and endothelial cells in the liver) as an inactive single-chain precursor (pro-HGF) which is bound to heparin proteoglycans within the extracellular matrix [11]. HGF transcription is upregulated by inflammatory modulators such as tumor necrosis factor alpha, IL-1, IL-6, TGF-beta, and VEGF [11, 12]. Circulating pro-HGF undergoes proteolytic conversion via extracellular proteases including HGF activator (HGFA), urokinase-type plasminogen activator, factors XII and XI, matriptase, and hepsin [8] into an active two-polypeptide chain heterodimeric linked by a disulfide bond. HGFA is a serine protease which is secreted primarily by the liver and circulates as pro-HGFA; pro-HGFA is activated by thrombin in response to tissue injury and malignant transformation [13, 14]. The active form of HGF includes an *α*-chain containing four kringle domains (K1 to K4) and an amino-terminal loop domain (N), and a *β*-chain (C-terminal) containing a serine protease homology (SPH) domain [12].

HGF is a ligand for the MET receptor, also known as cellular MET or cMET [15]. The *MET* protooncogene was first isolated in 1984 from a human osteosarcoma-derived cell line driven by a chromosomal rearrangement *TPR-MET*, resulting from fusion of *translocated promoter region* located on chromosome 1q25 and *MET* sequence located on chromosome 7q31 [16]. The *TPR-MET* rearrangement encodes for a prototype of the cMET receptor tyrosine kinase family. The cMET receptor is expressed predominantly on the surface of endothelial and epithelial cells of many organs, including the liver, kidney, prostate, pancreas, kidney, muscle, and bone marrow [7]. Like HGF, cMET is synthesized as an inactive single-chain precursor and undergoes proteolytic cleavage into a disulfide linked heterodimer consisting of an extracellular *α*-chain and transmembrane *β*-chain. The *β*-chain contains an extracellular domain, transmembrane domain, and cytoplasmic portion. The extracellular domain includes an amino-terminal semaphorin domain, terminal cysteine-rich PSI domain, and four IPT repeat immunoglobulin domains [17]. The cytoplasmic portion includes a juxtamembrane region including two phosphorylation sites, a tyrosine kinase (TK) domain and a carboxyl terminal region for substrate docking [18].

**Figure 1:**
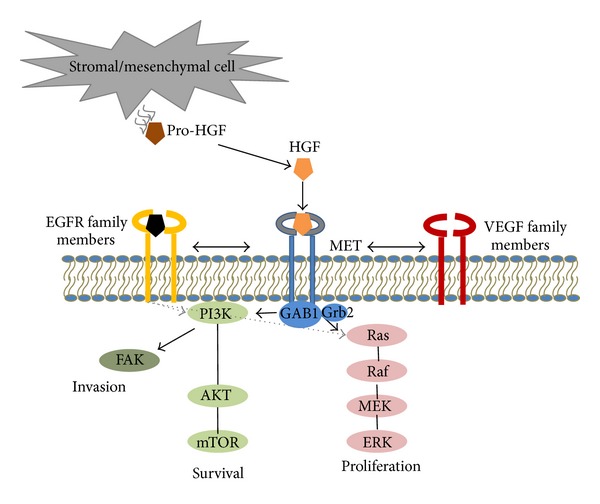
The hepatocyte growth factor-(HGF-) cMET axis. The hepatocyte growth factor (HGF) interacts with cMET via a paracrine signaling loop and mediates the epithelial-mesenchymal transition. Pro-HGF is secreted by stromal and mesenchymal cells and undergoes proteolytic activation into HGF. HGF binds to the MET receptor on epithelial cells and induces transautophosphorylation and binding of adaptor proteins. These provide scaffolding for recruitment of other signaling proteins and activation of signaling pathways resulting in increased invasion and motility, survival, proliferation, and stimulation of angiogenesis. Crosstalk between EGFR, cMET, and VEGF pathways is also implicated in promoting tumor survival. EGFR: epidermal growth factor receptor; FAK: focal adhesion kinase; Grb2: growth factor receptor-bound protein 2; GAB1: Grb2-associated binding protein; MEK, mitogen-activated protein kinase/ERK kinase; PI3K: phosphoinositide 3-kinase; mTOR: mammalian target of rapamycin; RAS: renin-angiotensin system; VEGF: vascular endothelial growth factor.

Under normal conditions, HGF interacts with cMET via a paracrine signaling loop and triggers cMET kinase activation within the cytoplasmic portion (see Figure 1). HGF contains two cMET binding sites including a high affinity constitutively active site on the *α*-chain (N-terminal and first kringle domain, or N-K1) and a low affinity site on the *β*-chain (C-terminal) [8, 19]. The exact mechanism of cMET receptor activation and the contribution of both binding sites to receptor activation by full length HGF are not yet established, although it is evident that both *α*- and *β*-chain sites have distinct functions. While *α*-chain N-K1 portion activates cMET and induces MET dimerization [20], *β*-chain residues bind cMET once the receptor is already occupied by HGF N-K4 and subsequently induce phosphorylation and downstream signaling [19].

HGF binding induces autophosphorylation of tyrosine residues Y1234 and Y1235 within the cMET TK domain activation loop. Phosphorylation also occurs at two sites within the carboxyl terminal region (Y1349 and Y1356), forming a multifunctional high affinity binding docking site that recruits a range of intracellular adaptors containing Src homology-2 domains [21–24]. An intact multifunctional docking site is necessary for malignant transformation. Recruited adaptor proteins and kinase substrates include growth factor receptor-bound protein 2 (Grb2), Grb2-associated binder (Gab1), phospholipase C-*γ*, STAT3, Shc, Src, Shp2, PI3K, and Ship1 [23, 24]. These and other adaptor proteins provide scaffolding for a larger apparatus of network proteins, ultimately promoting activation of multiple signaling pathways. Of these, Grb2 and Gab1 are critical effectors that interact directly with the receptor; Grb2 binding to the cMET docking site through Y1356 results in downstream signaling via the Ras/MAPK pathway, while Gab1 phosphorylation by MET kinase activates the PI3K pathway. Other signaling proteins that are activated by cMET include p38, JNK, and nuclear factor KB [13]. Alterations in transcription induce cell cycle progression, antiapoptosis, increased cell motility, angiogenesis, and survival.

The HGF-cMET pathway serves as a hub for multiple heterogeneous signaling networks and is also modulated by the activation of other receptor TK families such as the epidermal growth factor receptor (EGFR), human epidermal growth factor receptor 2, insulin-like growth factor 1 receptor, Raf kinase, and VEGF [25].

## 3. HGF-cMET Activation and HCC Pathogenesis

The HGF-cMET pathway plays an essential role in mammalian development in terms of morphogenesis, mitogenesis, and motogenesis, and angiogenesis HGF-cMET signaling is likely to be critical in many aspects of adult homeostasis including cardiac and hepatic tissue injury repair [26, 27], skin wound repair [28], and skin immunity regulation [29].

Targeted disruption of the *HGF* or *MET* genes results in embryonically lethal knockouts with impaired organogenesis of the liver and placenta [30]. Preclinical models demonstrate that HGF functions as a hepatotrophic factor enhancing hepatic regeneration and suppressing hepatocyte apoptosis [31, 32]; expression of HGF is increased in response to liver injury, while neutralization of endogenous HGF or *MET* knockout facilitates liver damage and fibrotic changes with delayed repair [8]. Under normal physiologic conditions, HGF-induced cMET activation is strictly controlled by paracrine ligand delivery followed by ligand activation at target cell surfaces and ligand-activated receptor internationalization/degradation [21]. Despite multiple checkpoints, HGF-cMET axis dysregulation occurs in a variety of solid tumors and hematopoietic derived malignancies and plays a key role in malignant transformation by promoting tumor cell migration, epithelial to mesenchymal transition, invasion, proliferation, and angiogenesis. Dysregulated cMET signaling upregulates protease production (plasminogen dependent and independent) and increased cell dissociation via extracellular matrix degradation, facilitating tumor invasiveness and metastasis [33]. Mechanisms of pathogenic activation include aberrant paracrine or autocrine ligand production, overexpression of HGF and cMET, upregulation of genes encoding proteases for HGF/cMET processing [24], constitutive kinase activation independent of *cMET* gene amplification, and *cMET* gene mutations leading to ligand-independent kinase activity [34–36].

The HGF-cMET axis is implicated in hepatocarcinogenesis through multiple mechanisms, many of which are still being elucidated. Overexpression of HGF [37] and cMET [37–41] is observed in 33% and 20–48% of human HCC samples, respectively. The prognostic utility of cMET and HGF overexpression is uncertain; while some studies show no correlation between cMET overexpression and tumor size or invasive behavior [38, 42] or HGF levels and survival [41], others demonstrate an inverse relationship with survival. Specifically, cMET overexpression was found to correlate with poorly differentiated HCC [43] and increased intrahepatic metastases along with decreased five-year survival [41]. After hepatectomy, cMET overexpression in HCC tissue has been correlated with early tumor recurrence, metastasis [44], and shorter 5-year survival [41, 45, 46]. A *cMET*-regulated gene expression signature was found to define a subset of human HCC with poor prognosis and aggressive phenotype and correlated with increased vascular invasion, increased microvessel density, and decreased mean survival time [47]. Higher HGF levels negatively correlate with survival per biomarker analysis of the SHARP trial [48] and prior data [49] and positively correlate with tumor size [50]; however given that HGF is secreted as an inactive precursor, overexpression alone is unlikely to guarantee pathway dysregulation. HGF has also been investigated as a potential biomarker for HCC development [51], but may also be a specific marker for liver cirrhosis [52]. 

The role of *MET* mutations and gene amplifications in HCC pathogenesis is unclear. While gains in 7q have been reported in HCC [53], gene amplification has not been reported as a significant source of increased cMET [11]. Somatic mutations have been observed in some cases of childhood HCC [54]. 

Crosstalk between cMET and EGFR [55, 56] and cMET and VEGF signaling pathways is also implicated in promoting tumor survival. cMET-cSrc has been shown to mediate EGFR phosphorylation and cell survival in the presence of EGFR inhibitors [57]. cMET is both an independent angiogenic factor and interacts with angiogenic survival signals promoted through VEGF. By upregulating hypoxia-inducible factor, hypoxia results in increased HGF expression in tumor and surrounding normal interstitial cells and increased MET expression in tumor and endothelial cells. HGF-cMET signaling induces upregulation of tumoral VEGF expression and endothelial VEGFR2 expression and downregulation of endogenous inhibitors of angiogenesis [58, 59]. Dual VEGF and cMET axis activity demonstrates increased capillary formation *in vivo*, tubulogenesis *in vitro*, and tumoral microvessel density [59]. 

## 4. Pharmacologic cMET Inhibitors

Given the predominant role of dysregulated HGF-cMET signaling in hepatocarcinogenesis, pharmacologic cMET inhibition is a promising therapeutic strategy. Targets for inhibition of the cMET signal transduction pathway include ligand-receptor interaction, cMET kinase activity, and cMET and adaptor protein interaction. HGF-cMET axis inhibitors can be broadly classified into biologic antagonists, c-MET adaptor protein inhibitors, small-molecule downstream pathway inhibitors, and small synthetic MET tyrosine kinase inhibitors (TKI). Of these, TKIs are the most common and the only ones to have completed phase 2 testing in HCC as of March 2013. Table 1 shows the selected HGF-cMET inhibitors in active clinical trials for either HCC or advanced solid malignancies (including HCC) as of March 2013. A comprehensive listing of HGF-cMET inhibitors in active clinical trials for all malignancies is maintained by the Bottaro NCI Lab and is available at https://ccrod.cancer.gov/confluence/display/CCRHGF/Home. 

Biologic antagonists inhibit cell surface interactions such as ligand-receptor binding or receptor clustering, preventing activation of downstream signaling. These include HGF competitive analogs, MET decoy receptor, and anti-HGF-cMET monoclonal antibodies. HGF competitive analogs compete with ligand for receptor binding without causing cMET dimerization and activation and include NK2 [60, 61], NK4 [62–64], and uncleavable HGF [65]; preliminary safety and drug development data in humans are pending. NK2 may be the least promising HGF competitive analog due to being a potent mitogen [66] and promoting metastases [67]. MET decoy receptors are soluble forms of the cMET extracellular domain which compete with HGF and inhibit cMET dimerization; *in vitro* and *in vivo* mice models demonstrate suppression of HGF-induced tumor cell migration and metastasis [68, 69]. Currently, uncleavable HGF and decoy MET have been evaluated in preclinical models only. Monoclonal antibodies targeting HGF and the extracellular domain of cMET are currently being explored in clinical trials (see Table 1), but data are not yet available for HCC. 

Given the importance of adaptor proteins in propagating downstream cMET signaling, cMET adaptor inhibitors offer unique potential for cMET specific inhibition [70]. As described above, cMET signaling is initiated through autophosphorylation of cytoplasmic tyrosines that form docking sites for adaptor proteins. Grb2 and Gab1 are critical effectors that interact directly with the cMET receptor, ultimately recruiting a larger apparatus of network proteins necessary for cMET signaling. Gab1 couples with cMET directly via docking site interaction, or indirectly through Grb2 [71]. Gab1 coupling with cMET requires Gab1 binding to the SH3 domain of Grb2, and cMET binding to the SH2 domain of Grb2 [72, 73]. C90 is a selective Grb2 antagonist with demonstrable inhibition of gastric cancer cell motility and matrix invasion *in vitro* and impaired metastatic spread of human prostate cancer cells *in vivo*; data in human studies have not been reported to date [74, 75]. 

Small-molecule downstream pathway inhibitors directed towards inhibition of STAT3 phosphorylation showed preliminary tolerability in a phase I trial of advanced solid tumors, but data are not yet available for HCC [76]. 

Synthetic small MET TKIs inhibit downstream signal transduction by preventing phosphorylation, either via competitive binding of the intracellular adenosine triphosphate (ATP) site in cMET's TK domain, or noncompetitive binding of a cMET region outside of the ATP binding site. While some of the TKIs in development are cMET specific, others target multiple pathways including VEGF, PDGFR, fms-related tyrosine kinase 3 (FLT3), v-kit feline sarcoma viral oncogene homolog protein (KIT), and anaplastic lymphoma kinase (ALK). Preclinical studies and clinical trials show tolerability and efficacy of cMET TKIs across a variety of solid malignancies. As of March 2013, promising results in the phase 2 randomized setting for HCC are available for two cMET inhibitors: tivantinib and cabozantinib. 

## 5. cMET Inhibitors and HCC

Tivantinib (ARQ 197) is an oral low-molecular-weight TKI which is non-ATP competitive. Safety and tolerability without drug-related worsening of hepatic function were previously reported in a phase Ib trial of 20 cirrhotic patients (Child-Pugh A and B) with HCC, with 2 or less prior systemic chemotherapy regimens [77]. Rimassa and colleagues reported results at ASCO 2012 of a phase II trial assigning 107 patients with unresectable HCC with ECOG PS 0-1 and Child-Pugh A in a 2 : 1 randomization to either second-line tivantinib or placebo with crossover allowed [78]. Although no difference in median OS occurred, the primary endpoint of time to progression (TTP) was met and favored tivantinib versus placebo (6.6 versus 6.2 months, HR = 0.90, *P* = 0.63; 6.9 versus 6 weeks, HR = 0.64, *P* = 0.04, resp.). Patients with high MET expression (defined as 50% or more cells in the tumor specimen with 2+ or 3+ staining intensity) versus low MET expression receiving tivantinib demonstrated a significant improvement in both OS and TTP (7.2 versus 3.8 months, HR 0.38 *P* = 0.01; 11.7 versus 6.8 weeks, HR 0.43, *P* = 0.03, resp.). No detrimental effect was reported in patients with low MET expression, and common adverse events were neutropenia, asthenia, poor appetite, and anemia. As of November 2012, a phase III trial for tivantinib and HCC patients is in development.

Cabozantinib (XL 184) is an unselective oral multikinase TKI targeting cMET, KIT, rearranged during transfection (RET), VEGFR1 and 2 and 3, FLT3, AXL receptor tyrosine kinase (AXL), and Tie family angiopoietin 1 receptor [79]. Cabozantinib has shown efficacy in the phase 3 setting for medullary thyroid carcinoma with progression-free survival (PFS) improvement and phase 2 setting for advanced solid tumors (including HCC). Verslype and colleagues reported preliminary results of a phase 2 randomized discontinuation study of cabozantinib in 41 patients with HCC, Child-Pugh score A, and one prior line of systemic treatment [80]. All patients initiated cabozantinib for a 12-week lead in time frame; patients with partial response continued on study drug while patients with progression of disease discontinued. 32 patients with stable disease were blindly randomized 1 : 1 to continue cabozantinib or receive placebo. The primary endpoint was overall response rate (RR) during the lead in time phase and PFS for patients entering the randomization phase. Independent of prior sorafenib use, median OS was 15.1 months, median PFS was 4.4 months, and median time on study was 6 months. Common grade 3 adverse effects were hand-foot syndrome, diarrhea, and thrombocytopenia. MET expression was not evaluated prospectively, and given the broad activity of cabozantinib, it is unclear how much activity is attributable to MET inhibition alone.

In fact, the inhibition of both VEGF and MET concurrently may be particularly effective. VEGF inhibition leads to increased MET signaling, either from resultant hypoxia or direct interactions between VEGFR2 and MET [81, 82]. Concurrent inhibition of cMET and VEGF suppresses tumor invasion and metastasis [82]. Preliminary evidence of efficacy against HCC was seen in a phase I combination study of tivantinib with sorafenib including a complete response and another prolonged partial response lasting greater than one year [83]. 

Table 1 notes ongoing trials with synthetic small MET TKIs. INC280 is a selective MET TKI in phase I testing for early HCC, currently accruing. Foretinib (GSK 1363089) is a small-molecule TKI targeting both MET and VEGF in phase I/II testing for advanced HCC. 

## 6. Patient Selection for HGF-cMET Pathway Inhibition 

Based on preclinical trials and phase 2 data for tivantinib and cabozantinib, inhibition of cMET signaling is a promising therapeutic strategy in HCC. Although it is unclear which genetic and molecular abnormalities implicated in HGF-cMET dysregulation are predictive of sensitivity to cMET targeted therapy, ongoing trials must address the challenge of identifying patients most likely to achieve maximal benefit and minimal toxicity. Two current strategies for patient selection based on tumor biomarkers are quantification of tumor cMET content via immunohistochemical (IHC) and immunoassay tissue analysis and assessment of MET sequence status. Pharmacodynamic serum markers are under evaluation in ongoing trials as a strategy to assess clinical response. Novel companion diagnostics are under evaluation in preclinical studies. 

Tumoral cMET overexpression as measured by commercially available IHC kits appears to correlate with efficacy of cMET inhibitors in HCC and other solid tumors, although more prospective data are necessary for validation. Identification of cMET phosphorylation status is a potentially powerful target for targeted antibodies; two site immunoassays of flash-frozen tissue samples yielding precise measurements of MET content and phosphorylation activation are currently under development [84]. 

Another promising stratification strategy is assessment of MET sequence status including *MET* mutations, *MET* amplification, and chromosome 7 polysomy. Preclinical studies of cMET targeted agents demonstrate variable efficacy based on *MET* mutation location; for example, PF-2341066/4217903 is a selective inhibitor with increased activity against certain cMET ATP binding site mutations in comparison to MET kinase domain activation loop mutations [85]. *In vitro* studies of SU11274, a small-molecule TK competitive ATP binding site inhibitor, show selective inhibition of two out of four identified *MET* mutations [86]. Amplification of *cMET* is associated with increased clinical response to foretinib (XL 880) in phase 2 gastric cancer data, and *cMET* copy number correlates with increased clinical response to tivantinib in addition to erlotinib in advanced NSCLC [87]. 

Ancillary pharmacodynamic and pharmacokinetic marker studies in a variety of solid tumors show variable correlation with clinical response. Preclinical data with crizotinib in gastric cancer cell lines demonstrate variable biomarker modulation of cMET inhibition with cMET dependent gastric cancer cell lines versus cMET-independent lines [88, 89]. Clinical data shows changes in plasma concentrations of HGF, VEGF, soluble MET, soluble VEGFR2, PIGF, and EPO during treatment with various TKIs [90, 91]. The predictive utility of these biomarkers in patients with cMET-dependent HCC is unclear; further analyses and prospective validation are necessary. 

HGF-cMET is an intriguing target for the development of companion diagnostic tools as an adjunct tool for patient selection and stratification for cMET therapy. *In vitro* and *in vivo *animal studies suggest that radiolabelled dyes containing cMET binding peptide successfully targets cMET receptors with higher imaged based tumor uptake [92]. A variant of SU11274 with radiomethylation modification is being utilized as a PET visualization agent to quantify cMET receptor in xenograft models [93]. 

## 7. Conclusion and Future Directions

Inhibition of cMET is a promising therapeutic strategy in HCC. Given the heterogeneous mechanisms underlying cMET dysregulation, there is an urgent and unmet need for the development of predictive biomarkers to identify which subsets of cMET-dependent HCC tumors are most likely to benefit from specific classes of inhibitors. As more agents move into phase 2 and 3 trials for HCC, one important consideration is the emergence of acquired and primary resistance mechanisms from de novo or preexisting mutations. These may be overcome by rational combination therapy directed against multiple pathways, different levels of ligand-receptor-TKI interaction, and the presence of cMET addiction. Innovative clinical trial designs (such as the discontinuation cabozantinib design) with incorporation of enriched patient cohorts, biomarker analyses, pharmacodynamic markers, and companion diagnostics are essential in moving forward.

## Figures and Tables

**Table 1:** HGF-cMET inhibitors in active clinical trials for HCC or solid tumors including HCC, as of March 2013.

Agent	Type	Drug targets	Phase	Patient selection	Status	NCI reference	Manufacturer
ARQ 197							
Tivantinib (T) T plus sorafenib T plus pazopanib T plus temsirolimus T plus topotecan	Receptor TKI: non-ATP competitive	cMET	I I Ib I I	HCC Solid tumors Solid tumors* Solid tumors Solid tumors	Recruiting Active, not R Recruiting Recruiting Recruiting	NCT01656265 NCT00827177 NCT01468922 NCT01625156 NCT01654965	Daiichi Sankyo

XL 184							
Cabozantinib	Receptor TKI: ATP competitive	cMET, RET, VEGFR1-3, KIT, FLT3, and TIE2	II II	Solid tumors Solid tumors	Active, not R Recruiting	NCT00940225 NCT01588821	Exelixis

INC280							
Formerly INCB028060	Receptor TKI: ATP competitive	cMET	I I I	Solid tumors Solid tumors* Solid tumors	Recruiting Recruiting Active, not R	NCT01546428 NCT01324479 NCT01072266	Novartis Incyte

GSK1363089							
Foretinib Formerly XL 880	Receptor TKI: ATP competitive	cMET, RON, VEGFR1-3, PDGFR, KIT, FLT3, and TIE2	I/II	HCC	Active, not R	NCT00920192	GSK

AMG 208	Receptor TKI: ATP competitive	cMET, VEGFR1-3, RON, and TIE2	I	Solid tumors	Active, not R	NCT00813384	Amgen

AMG 337	Receptor TKI: ATP competitive	cMET	I	Solid tumors	Recruiting	NCT01253707	Amgen

EMD 1214063	Receptor TKI: ATP competitive	cMET	I	Solid tumors*	Recruiting	NCT01014936	EMD Serono

PF-02341066							
Crizotinib (Cr) Crizotinib Cr plus pemetrexed or pazopanib	Receptor TKI: ATP competitive	cMET, ALK, and ROS	I I I	Solid tumors* Solid tumors Solid tumors*	Recruiting Recruiting Recruiting	NCT00585195 NCT01576406 NCT01548144	Pfizer

E7050							
E7050 E7050 plus sorafenib	Receptor TKI: ATP competitive	cMET	I I/II	Solid tumors HCC	Recruiting Recruiting	NCT01428141 NCT01271504	Eisai

MGCD-265							
MGCD-265 MGCD-265 plus erlotinib or docetaxel	Receptor TKI: ATP competitive	cMET, RON, VEGFR1-2, PDGFR, KIT, FLT3, and TIE2	I I/II	Solid tumors Solid tumors	Recruiting Recruiting	NCT00697632 NCT00975767	MethylGene

SAR125844	Receptor TKI: ATP competitive	cMET	I I	Solid tumors* Solid tumors*	Recruiting Recruiting	NCT01391533 NCT01657214	Sanofi

ASLAN002							
Formerly BMS777607	Receptor TKI: ATP competitive	cMET	I	Solid tumors	Recruiting	NCT01721148	Aslan

AV-299							
Ficlatuzumab plus/minus erlotinib Formerly SCH 900105	Ligand antagonist: monoclonal antibody	HGF	I	Solid tumors	Active, not R	NCT00725634	AVEO

LY 2875358							
LY 2875358 LY 2875358 plus/minus erlotinib	Receptor inhibitor: monoclonal antibody	cMET	I I	Solid tumors Solid tumors	Recruiting Recruiting	NCT01602289 NCT01287546	Eli Lilly

OPB-31121	IL-6-induced STAT3 phosphorylation inhibitor	STAT3	I/II	HCC	Recruiting	NCT01406574	Otsuka

OPB-51602	IL-6-induced STAT3 phosphorylation inhibitor	STAT3	I I	Solid tumors Solid tumors	Active, not R Recruiting	NCT01423903 NCT01184807	Otsuka

*Inclusion criteria require evidence of cMET dysregulation for some or all patients.

Active, not R: active not recruiting; ALK: anaplastic lymphoma kinase; ATP: adenosine triphosphate; GSK: GlaxoSmithKline; HGF: hepatocyte growth factor; HCC: hepatocellular carcinoma; IL6: interleukin-6; PDGFR: platelet-derived growth factor receptor; STAT: signal transducer and activator of transcription; TKI: tyrosine kinase inhibitors; and VEGFR: vascular endothelial growth factor receptor.
